# Long-term efficacy of sialendoscopy in treating childhood Sjögren’s disease with chronological monitoring by salivary gland ultrasonography: A novel approach

**DOI:** 10.1186/s12969-023-00870-3

**Published:** 2023-08-13

**Authors:** Kristin Drew, René Kronlage, Seunghee Cha, Akaluck Thatayatikom, Thomas Schrepfer

**Affiliations:** 1 College of Medicine, University of Florida, Gainesville, FL USA; 2 Department of Oral & Maxillofacial Diagnostic Sciences, University of Florida, Gainesville, FL USA; 3 Department of Pediatric Rheumatology, Advent Health, Orlando, FL USA; 4 Department of Otolaryngology (ENT), Otolaryngology Head and Neck Surgery, College of Medicine, University of Florida, 1345 Center Drive, MSB, M2-228, Box 100264, Gainesville, FL 32610 USA

**Keywords:** Childhood Sjögren’s Disease, Recurrent parotitis, Pediatric, Salivary gland Ultrasonography (SGUS), Sialendoscopy, Sicca symptoms

## Abstract

**Background:**

Childhood Sjögren’s Disease (cSjD) is an underdiagnosed phenomenon with clinical and pathophysiological nuances in contrast to Sjögren’s Disease (SjD) in the adult population. While adults typically experience sicca symptoms, children with cSjD often present with recurrent parotitis, diverse autoantibody profiles, and renal and neurological manifestations. Diagnosis and classification in pediatric rheumatology remain controversial due to the reliance on adult-focused diagnostic criteria and the lack of standardized treatment and understanding of outcomes. The purpose of the paper is to propose a multimodal treatment plan and demonstrate the effectiveness of sialendoscopy in the management of cSjD.

**Case Presentation:**

We present the case of a twelve-year-old female diagnosed with cSjD using the 2016 American College of Rheumatology (ACR) and the European League Against Rheumatism (EULAR) diagnostic criteria for SjD. In addition to medical management, she underwent sialendoscopy with triamcinolone irrigation under sedation and was monitored for progress via salivary gland ultrasonography (SGUS). Over the course of one year, she demonstrated significant improvement in symptoms, with serial SGUS scores gradually decreasing by five points.

**Conclusions:**

This paper proposes a multimodal treatment plan involving sialendoscopy and medical management as a non-invasive and potentially more effective approach for cSjD. Standardized monitoring through SGUS scoring allows objective and quantifiable measurement of treatment progress, enabling better assessment of glandular tissue status. Recurrence is possible, and each cSjD patient may present differently. Nevertheless, our year-long observation of a patient with cSjD demonstrates that sialendoscopy, as seen in adults, can promote remission of recurrent parotitis in children as well.

## Introduction

Sjögren’s Disease (SjD) is an autoimmune inflammatory condition that presents as a chronic, multisystem disease, often affecting women between the ages of 45–55 years of age. Characterized by immune-mediated exocrine gland destruction, adults with SjD often report sicca symptoms of xeropthalmia and xerostomia [1]. However, in contrast to the adult population, childhood Sjogren’s Disease (cSjD) more commonly presents with recurrent parotitis, diverse autoantibody profiles, and renal and neurological manifestations [2, 3]. Aside from clinical presentation, differences in the pathophysiology between SjD and cSjD are evident in the evaluation of glandular histology. In children with cSjD, histopathology often shows punctate sialectasis, but adults with SjD will demonstrate fatty infiltration within their glandular tissue [4].

In 2016, the American College of Rheumatology and European League Against Rheumatism (ACR/EULAR) set forth criteria for the diagnosis of SjD in the adult population. According to the ACR/EULAR, a diagnosis of SjD is made through a point system in evaluation of the following clinical factors: serology detecting anti-SSA/Ro (SSA), focus score from labial biopsy, sialometry results for unstimulated saliva flow rate (USFR), Schirmer’s test, and ocular surface staining (OSS) [5]. Positive SSA and labial biopsy are each assigned a weight of three points, and USFR, Schirmer’s test, and OSS are each assigned one point. A SjD diagnosis is made if a patient presents with four or more points [5, 6]. It should be noted that sicca symptoms are not factored into diagnostic evaluation due to subjectivity [6].

Children with cSjD often do not meet the ACR/EULAR criteria, and many authors have proposed creating more specific criteria for the diagnosis of cSjD. In addition to differing clinical presentations, other barriers can exist in evaluating the pediatric population. According to Thatayatikom et al., the documentation of sicca symptoms in children can vary depending upon a child’s ability to communicate any sensation of xerostomia or xerophthalmia [7]. Furthermore, several diagnostic tests commonly used to identify SjD in adults may not be practical or lack established normative values adjusted for age in children [1]. Due to this limitation, salivary gland ultrasonography (SGUS) has been proposed as a highly sensitive and non-invasive addition to the ACR/EULAR criteria. Currently, along with SGUS, sialendoscopy is not recognized as standard of care treatment for patients with cSjD within the ACR/EULAR criteria [5, 8]. Since the diagnosis, classification, and treatment of cSjD remains controversial, the authors of this paper propose a multimodal therapeutic approach. We propose that patients with recurrent parotitis secondary to cSjD will benefit from the addition of sialendoscopy with triamcinolone irrigation. In addition, patients should concurrently undergo serial SGUS imaging with scoring to not only observe anatomical changes but also quantify results over time.

## Case report

**Fig. 1:**
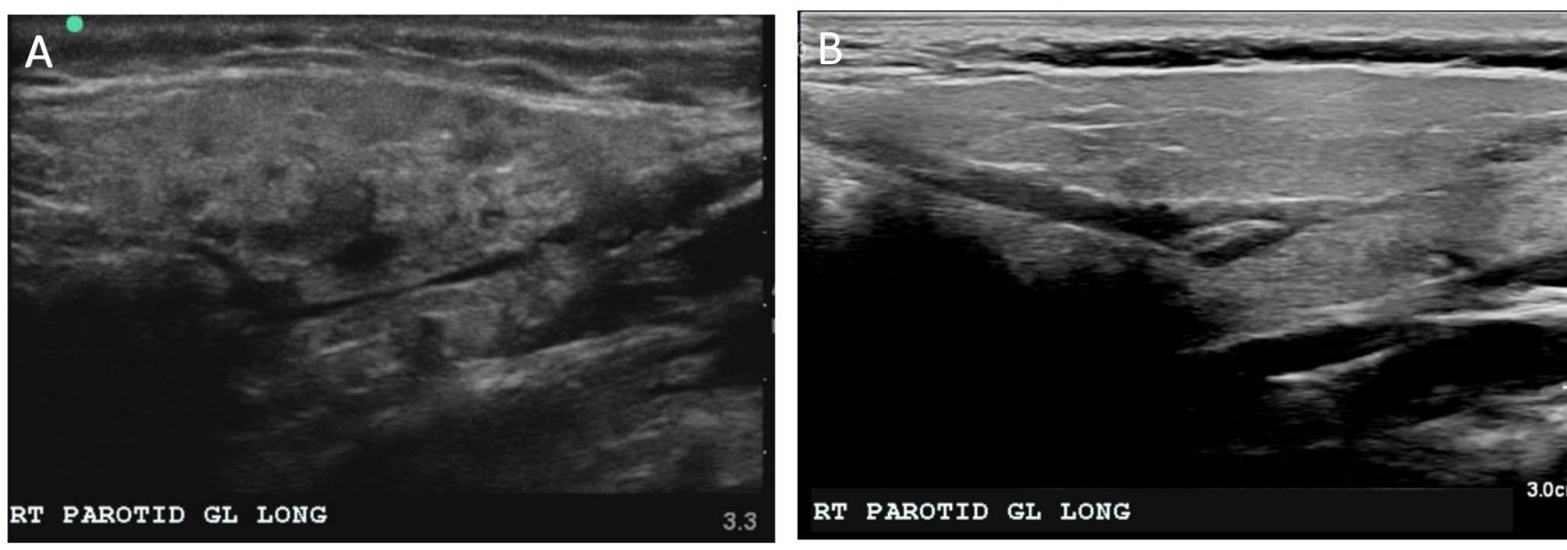
Right parotid ultrasound at baseline before sialendoscopy with SGUS score of 16 (A) in comparison with a healthy individual of same age (B)

**Fig. 2:**
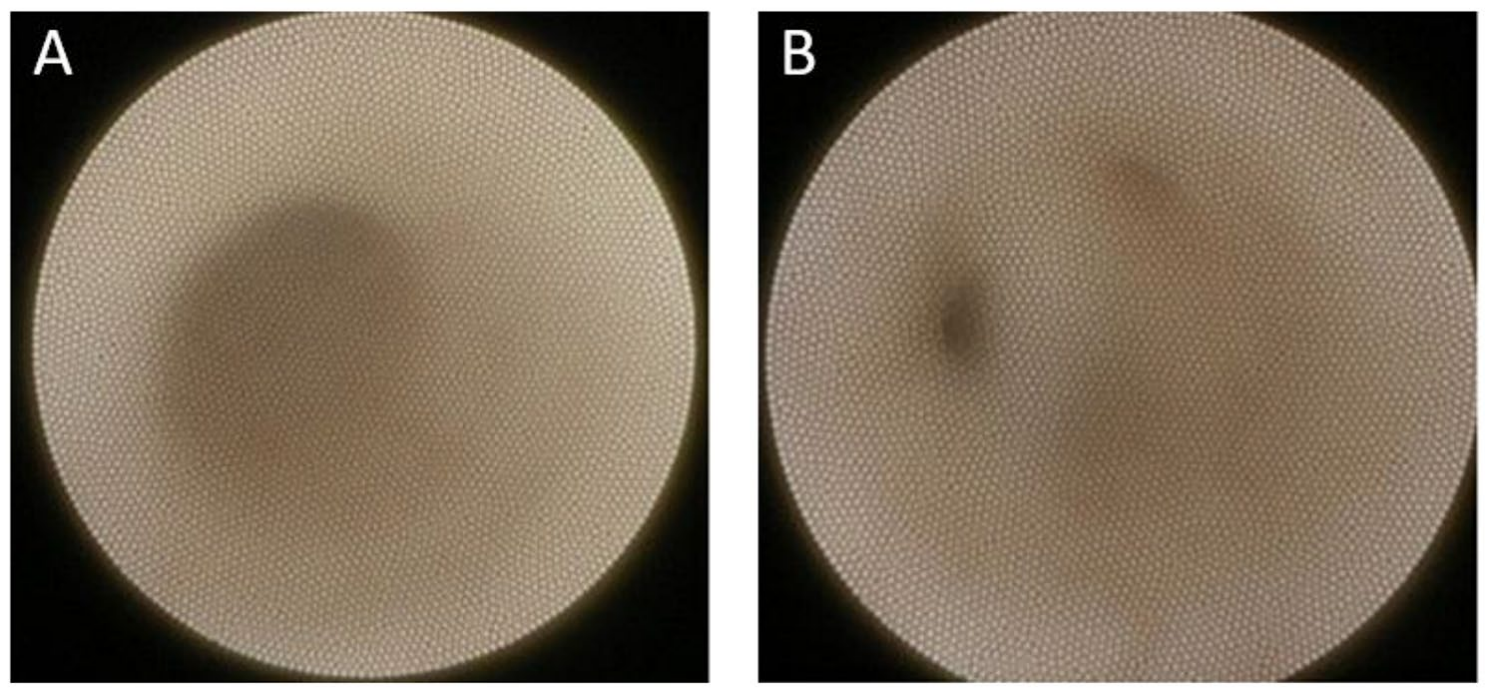
Sialendoscopic image of the Stensen’s duct (A) with its intraparotid bifurcations (B), which presented with a somewhat diffuse narrowing and white appearing inner lining

A twelve-year-old female with a history of type I diabetes mellitus presented with recurrent, tender parotitis refractory to antibiotics, xerostomia, and keratoconjunctivitis sicca since the age of five. Her parotitis was more pronounced on the right side, with the inflammation resulting in mild otalgia. Her lab work was negative for SSA (Ro) and SSB (La) autoantibodies, but positive for salivary protein 1 IgA autoantibody and antinuclear antibody (ANA). She also tested positive for labial biopsy with a focus score greater than one, and her sialometry measured below the cut-off for hyposalivation (0.099 ml/min). Her clinical symptoms, serologic markers, positive lip biopsy, and sialometry measurements warranted a formal diagnosis of childhood Sjögren’s Disease (cSjD). She underwent salivary gland ultrasonography (SGUS), with scoring criteria as proposed by Hocevar et al. [9] Her baseline SGUS score was 16, and imaging revealed multiple hypoechoic lesions in each parotid gland (Fig. 1). Ophthalmologic exam showed normal tear break up time, small tear lake, blepharitis, and mild hyperopia. Schirmer’s test was attempted, but the patient could not tolerate the test. Pharmacologic treatment included 200 mg hydroxychloroquine daily, and she underwent bilateral parotid duct and gland sialendoscopy with 100 mg triamcinolone irrigation to each parotid gland under sedation. Upon visualization of the ductal system the inner lining appeared white, indicating a lack of vascularity. While patency was maintained without obvious obstruction, some diffuse narrowing was present (Fig. 2).

**Fig. 3:**
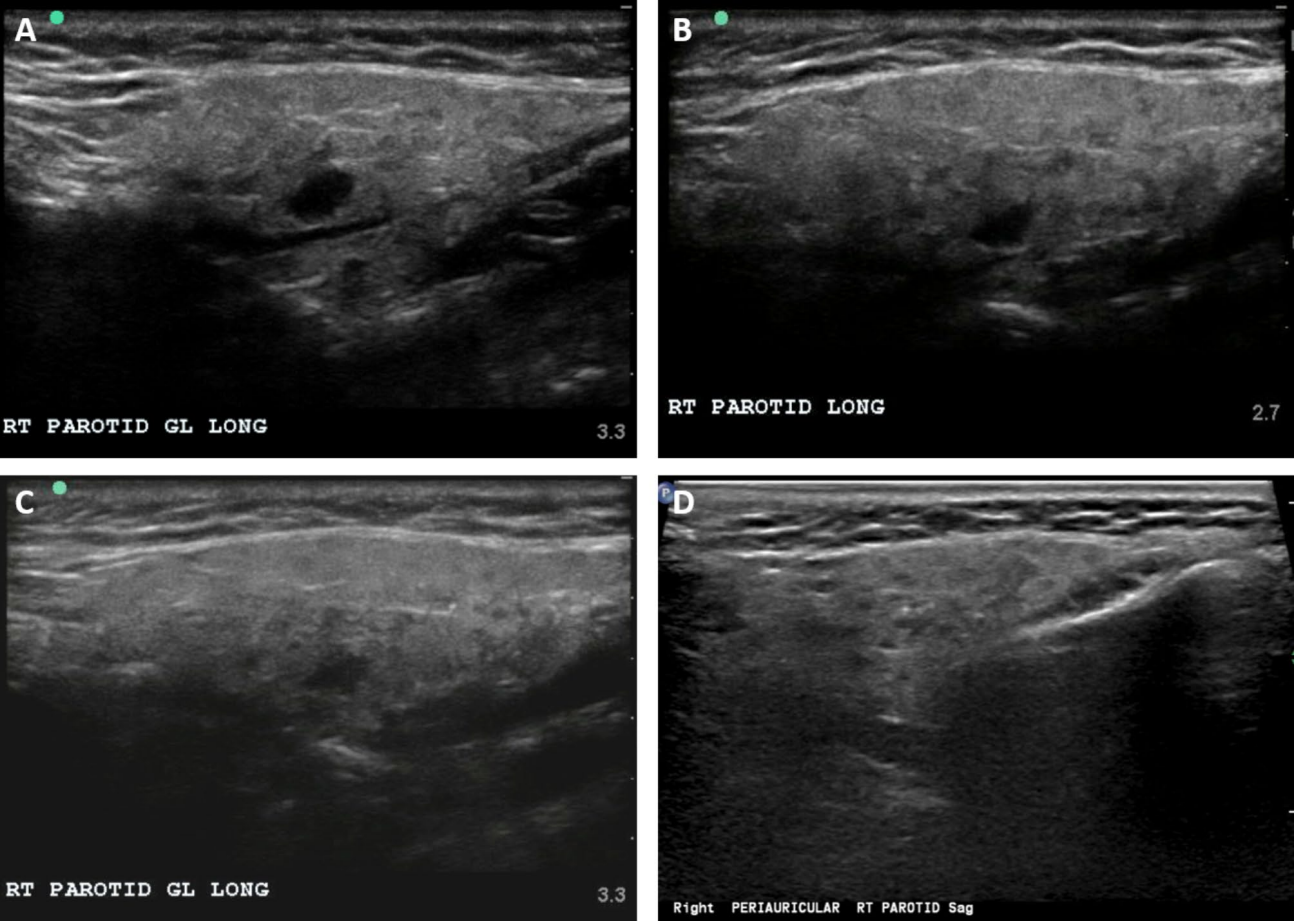
Right parotid gland at three months post-sialendoscopy with a SGUS score of 10 (A), four months post-sialendoscopy with a SGUS score of 11 (B), six months post-sialendoscopy with a SGUS score of 11 (C) and at 12 months post-sialendoscopy with a SGUS score of 11 (D)

In the months following sialendoscopy, her SGUS score decreased to 10 or 11 at all subsequent appointments, indicating significant improvement in glandular inflammation (Fig. 3). The imaging revealed homogeneous findings, suggesting reduced inflammation. Six months after sialendoscopy, sialometry measurements still showed a flow rate below 0.1 mL/min. Eleven months post-sialendoscopy, the patient experienced recurrent swelling in the right parotid gland. As a precautionary measure, the hydroxychloroquine dose was increased to 300 mg, and methylprednisolone was prescribed as needed for symptom exacerbation. However, the patient reported not requiring systemic steroid treatment during that time. Twelve months after sialendoscopy, she reported no symptoms, clinical examination showed no parotid swelling, bilateral expression of clear saliva was observed, and the SGUS score remained at 11 (Fig. 3).

## Discussion

### Sialendoscopy

Few reports adequately estimate the effectiveness of sialendoscopy in halting recurrence of childhood Sjögren’s Disease (cSjD). Sialendoscopy is a safe, minimally invasive procedure which can be effective for addressing salivary issues in SjD. Benefits include direct visualization of ductal pathologies, including inflammation, stenosis, and stricture diagnosis. In addition to gathering images, the physician can also administer a treatment when indicated [10]. The impact of sialendoscopy has been studied extensively in the adult population with SjD. However, to our knowledge, Iorio et al. are the only authors who have explored the impact of sialendoscopy and steroid irrigation on children [11].

Currently, no guidelines establish sialendoscopy with irrigation as part of the standard of care for treatment, as both cSjD and SjD are often medically managed with anti-inflammatory treatments or immunomodulatory therapy. When reviewing outcomes in patients with recurrent juvenile parotitis, Schwarz et al. determined it is unclear whether steroid administration or sialendoscopy itself relieves severity of inflammatory symptoms [10]. Du et al. performed a randomized controlled trial assessing the efficacy of sialendoscopy itself by comparing outcomes in adults with SjD between three treatments: those who did not undergo sialendoscopy, patients who received saline irrigation, and patients who received irrigation with triamcinolone acetate [12]. Patients who underwent sialendoscopy, either with irrigation of saline or triamcinolone, showed significant improvement in salivary flow at one to eight weeks. However, the patients who received the triamcinolone treatment continued to experience consistent improvement in salivary flow at 16 to 24 weeks. Izumi et al. also noted that their patients who received corticosteroids showed greater efficacy in improving salivary flow rate in comparison to patients who started with saline irrigation [13]. Thus, administration of triamcinolone appears to show clinically significant results in reducing inflammation.

The 2002 American-European Consensus Group (AECG) criteria [14], 2012 American College of Rheumatology (ACR) classification criteria [15], and the 2016 ACR/EULAR criteria [6] do not incorporate sialendoscopy as a factor for the diagnosis or treatment of cSjD. With that said, many clinicians utilize sialendoscopy for the management of inflammatory parotitis. Faure et al. noted that sialendoscopy has a greater sensitivity than radiologic ultrasound or MRI, and Schiffer et al. have established that cSjD diagnoses can be based upon sialendoscopy when used in combination with histopathology from labial biopsy [16–18]. While sialendoscopy offers an exceptional diagnostic and treatment modality, it is important to note that it is not sufficient for making a definitive diagnosis or providing a cure for all salivary gland disorders, particularly those resulting from autoimmune inflammatory processes. Therefore, clinical indicators of cSjD, as outlined by the ACR/EULAR criteria, should be included when determining a diagnosis. Patients with cSjD and autoimmune disorders are at a higher risk of symptom recurrence compared to those with obstructive salivary gland disorders, necessitating continued monitoring and follow-up examinations after treatment.

Patients diagnosed with cSjD present with a unique pathophysiology of the parotid glands in comparison to the adult population. Karagozoglu noted that the improvement of salivary flow is most likely dependent upon the presence of saliva-producing acinar cells, which may be lacking in patients with fibrotic tissue, secondary to long-standing disease [19]. Therefore, pediatric patients with a recent onset of cSjD and increased salivary gland capacity would likely benefit from undergoing sialendoscopy, as their tissue would be less likely to exhibit fibrosis and resulting fatty infiltration.

Iorio et al. evaluated the long-term impacts of sialendoscopy with corticosteroid irrigation in children aged 6 to 13, and improvement in salivary flow was noticeable during an 18-month observation period [11]. The biopsy report of our patient indicates normal glandular architecture throughout, without evidence of fibrotic change with normal-appearing acini, fulfilling the original guideline for SjD histological evaluations [20]. Based on the results of our patient, along with the results of Iorio et al., further investigation comparing the effects of sialendoscopy between children and adults is warranted, considering the unique pathophysiology of cSjD and SjD, respectively.

Numerous studies have evaluated the efficacy of sialendoscopy over different timeframes. Karagozoglu et al. conducted follow-ups with patients for up to sixty weeks after undergoing sialendoscopy with triamcinolone acetate [21]. Their results demonstrated a significant increase in salivary flow, indicating long-term efficacy considering the typical disease course. In a study by Du et al., saline provided immediate relief similar to triamcinolone at one to eight weeks, but patients who received triamcinolone irrigation showed continued improvement in salivary flow rate at sixteen and twenty-four weeks [12].

Our patient experienced recurrence of right-sided parotitis at eleven months. However, overall improvement was noted over 48 weeks after her hydroxychloroquine dosage was increased to 300 mg. While cSjD is a rare condition, recurrence is not uncommon in other cases. Iorio reported recurrences of parotitis in two patients at five and three years. It is important to note that sialendoscopy is a treatment modality for managing salivary gland disorders and is focused on addressing specific concerns related to the salivary glands. Therefore, the optimal treatment plan for a patient with cSjD should remain multifactorial, encompassing both medical and procedural management, as each patient with an autoimmune condition presents uniquely with their own respective clinical presentation.

### Salivary gland Ultrasonography

In 2005, Hocevar et al. set forth a novel scoring system through SGUS. Through SGUS, the inflammation of glandular tissue is quantified via observation of parenchymal echogenicity in comparison to the thyroid, homogeneity of tissue parenchyma, hypoechoic areas, hypoechoic foci, and delineation [9]. According to Hocevar et al., a score of 17 or greater on a scale ranging from 0 to 48 indicates a diagnosis for Sjögren’s Disease (SjD), along with consideration of the broader clinical picture and plasma titers. Theander and Mandl note that SGUS is an effective method for diagnostic and prognostic purposes in diagnosing SjD, as it has a positive predictive value and specificity of 98% [22].

In our patient, SGUS was utilized to offer not only a visual imaging but also provide a quantified score in assessing efficacy of treatment post-sialendoscopy. Our patient’s SGUS score decreased from 16 to 11, and she reported being asymptomatic without flares after one year. Her clinical improvement was reflected by subsequent SGUS results and a normal physical exam upon follow-up. We conclude SGUS is best utilized to monitor each patient’s individual progress via comparison of imaging and scoring with the first established baseline.

Notably, our patient presented with a baseline score of 16 in her SGUS, which is one point below the cutoff for Hocevar for SjD. With that said, half of the subjects with SjD in Hocevar et al’s study scored below 17 in their SGUS assessment. In addition, in Hocevar’s study all subjects were adults between the ages of 23–84 with a mean age of 51.5. As discussed, cSjD presents with unique pathophysiology. Therefore, further investigation is warranted on whether the cutoff or parameters for a diagnosis of cSjD via SGUS should be modified for the pediatric population. As seen in our patient, a reduction in five points suggested a curtailment of inflammatory processes within her parotid tissue. However, the degree of point reduction should be compared to more patients with cSjD who have undergone sialendoscopy. In addition, a modified SGUS for the pediatric population might reflect a greater difference in scoring.

It should be noted that SGUS does present with other limitations. Drawbacks can include missing early disease if disease severity is mild, and SGUS monitoring should be executed with the same technique, machine, technician, and standardized scoring system if long-term follow-up is planned. Since cSjD is a systematic inflammatory disease process, clinicians should also continue to monitor renal, neurologic, and musculoskeletal function in a thorough workup.

## Conclusion

Along with medical management, sialendoscopy can be utilized as a therapeutic treatment with low morbidity for treatment of parotitis secondary to cSjD. Its success in the treatment of inflammatory processes is likely due to the dilation of the ductal system under direct visualization in combination with corticosteroid irrigation. Further studies are warranted over whether the addition of sialendoscopy can provide improved long-term remission of recurrent parotitis in patients with cSjD. As no criteria exist for diagnosing pediatric patients with cSjD, it is our opinion that sialendoscopy and SGUS can be beneficial when incorporated in the management of cSjD, especially when a patient presents with recurrent parotitis.

## Data Availability

Data supporting this study are included within the article and/or supporting materials.

## Abbreviations

ACR: American College of Rheumatology
AECG: American-European Consensus Group
cSjD: childhood Sjögren’s Disease
EULAR: European League Against Rheumatism
OSS: ocular surface staining
SGUS: salivary gland ultrasonography
SjD: Sjögren’s Disease
USFR: unstimulated saliva flow rate
